# Glucocorticoid-Induced Leucine Zipper (GILZ) in Cardiovascular Health and Disease

**DOI:** 10.3390/cells10082155

**Published:** 2021-08-21

**Authors:** Donato Cappetta, Oxana Bereshchenko, Eleonora Cianflone, Francesco Rossi, Carlo Riccardi, Daniele Torella, Liberato Berrino, Konrad Urbanek, Antonella De Angelis, Stefano Bruscoli

**Affiliations:** 1 Department of Experimental Medicine, University of Campania ‘Luigi Vanvitelli’, 80138 Naples, Italy; donato.cappetta@unicampania.it (D.C.); francesco.rossi@unicampania.it (F.R.); liberato.berrino@unicampania.it (L.B.); 2 Department of Philosophy, Social Sciences and Education, University of Perugia, 06123 Perugia, Italy; oxana.bereshchenko@unipg.it; 3 Department of Medical and Surgical Sciences, University ‘Magna Graecia’ of Catanzaro, 88100 Catanzaro, Italy; cianflone@unicz.it; 4 Department of Medicine and Surgery, Section of Pharmacology, University of Perugia, 06156 Perugia, Italy; carlo.riccardi@unipg.it (C.R.); stefano.bruscoli@unipg.it (S.B.); 5 Department of Experimental and Clinical Medicine, University ‘Magna Graecia’ of Catanzaro, 88100 Catanzaro, Italy; dtorella@unicz.it (D.T.); urbanek@unicz.it (K.U.)

**Keywords:** glucocorticoid-induced leucine zipper, glucocorticoids, cardiovascular disease, inflammation

## Abstract

Glucocorticoids (GCs) are essential in regulating functions and homeostasis in many biological systems and are extensively used to treat a variety of conditions associated with immune/inflammatory processes. GCs are among the most powerful drugs for the treatment of autoimmune and inflammatory diseases, but their long-term usage is limited by severe adverse effects. For this reason, to envision new therapies devoid of typical GC side effects, research has focused on expanding the knowledge of cellular and molecular effects of GCs. GC-induced leucine zipper (GILZ) is a GC-target protein shown to mediate several actions of GCs, including inhibition of the NF-κB and MAPK pathways. GILZ expression is not restricted to immune cells, and it has been shown to play a regulatory role in many organs and tissues, including the cardiovascular system. Research on the role of GILZ on endothelial cells has demonstrated its ability to modulate the inflammatory cascade, resulting in a downregulation of cytokines, chemokines, and cellular adhesion molecules. GILZ also has the capacity to protect myocardial cells, as its deletion makes the heart, after a deleterious stimulus, more susceptible to apoptosis, immune cell infiltration, hypertrophy, and impaired function. Despite these advances, we have only just begun to appreciate the relevance of GILZ in cardiovascular homeostasis and dysfunction. This review summarizes the current understanding of the role of GILZ in modulating biological processes relevant to cardiovascular biology.

## 1. Introduction

Stress is a well-recognized factor in cardiovascular diseases [1]. According to the first definition of stress by Hans Selye in 1936 as “the non-specific neuroendocrine response of the body” [2], all stimuli are “stressors” that produce a general response regardless of the stressors’ nature, resulting in the unbalanced homeostasis of the organism [3]. In recent decades, a myriad of studies have confirmed the role of biological stress (oxidative, hemodynamic, metabolic, etc.) in cardiovascular disease, and have linked cardiovascular risk factors to chronic stress. Furthermore, mental and emotional stress may determine the course towards cardiovascular disease [4].

Chronic exposure to stressors causes endocrine and immune dysregulation that sustains low-grade inflammation, which evolves to become systemic and detrimental. The endocrine system responses to stress by producing and secreting hormones that regulate a wide range of biological functions, including growth and development, sexual reproduction, metabolism, heart rate, blood pressure, sleeping and waking cycles, etc. [5,6]. GCs are also called “stress hormones”, and their increased secretion upon anxiety and severe injury is desirable, but when chronically elevated, circulating GCs are linked to adverse cardiovascular outcomes [7,8]. The role of GCs in maturation of the fetal heart further supports the importance of GC signaling in cardiovascular biology [9,10].

The purpose of this review is to outline the possible role of GC-induced leucine zipper (GILZ) in the cardiovascular system, accentuating its contribution to the adaptations of stressed myocardium and the vascular response upon chronic stress. Although the available data are limited at present, the role of this GC-target protein possibly extends beyond its anti-inflammatory/immunosuppressive effects.

Herein, a section that highlights GC signaling and its role in the cardiovascular system was kept synthetic as these arguments are extensively reviewed elsewhere [6,11].

## 2. Glucocorticoid Signaling

GCs are a highly conserved family of steroid hormones released upon the activation of the hypothalamic-pituitary-adrenal axis. In response to stress, cortisol is secreted by adrenal glands into the bloodstream, through which it reaches tissues and organs where it coordinates physiological processes such as energy metabolism, resolution of inflammation, sperm maturation, and endocrine functions [6,12,13,14].

Physiological and pharmacological actions of endogenous and exogenous GCs are mediated by the GC receptor (GR), a member of the nuclear receptor superfamily of transcription factors, which in its inactive state is confined within the cytoplasm, bound to heat shock proteins and immunophilins (including hsp90, hsp70, FKBP52) [15]. The genomic mechanisms of transcriptional control operate in the nucleus. After binding, the ligand-receptor complex translocates to the nucleus in its dimeric form where it binds to specific DNA sequences, known as GC response elements, activating or suppressing gene transcription, thus mediating most of the GC effects. Increased gene transcription is mostly determined by homodimerization of the activated GR and its binding to DNA regulatory regions called GC-responsive elements (GREs), while decreased gene transcription results from heterodimerization of activated GR with other transcription factors, thus inhibiting their transcriptional activity. Another important mechanism by which GCs indirectly decrease gene transcription is the upregulation of inhibitors of transcription factors or the interaction with negative GREs [16]. Non-genomic effects are mainly characterized by a short delay of action and often involve modulation of intracellular signal-transduction cascades, such as phospholipases, cyclic AMP, protein kinases and calcium mobilization [17]. Actinomycin-D or cycloheximide, which are inhibitors of protein synthesis, did not revert the non-genomic effects, thus suggesting that these effects of GCs do not involve protein synthesis and hence are very rapid. These effects require GC/GR interaction, suggesting that GR most liley has other functions apart from its classic role as transcription factor. These rapid GC effects may also affect the cardiovascular system; for example, GR activation has been linked to rapid biological reduction in contractility, vascular reactivity, and endothelial nitric oxide synthase (eNOS) activation [18]. On the other hand, GR may interact with many kinases or transcription factors in the cytosolic compartment (i.e., nuclear factor-κB (NF-κB) or PI3K-Akt), so that direct and indirect transcription regulation is consequent to GC treatment [19,20,21,22].

The GR consists of various portions: in the *N*-terminal end is present the transactivation domain; the central section of the polypeptide chain is highly conserved (containing zinc fingers motifs necessary to bind DNA), while the *C*-terminal portion allows binding of the ligand. In humans, the gene for GR is located on the long arm of chromosome 5, is composed of 9 exons and, since exon 9 can be incorporated into the transcript in an alternative way, it serves as a template for at least two mRNAs: GR-α and GR-β. GR-α consists of 777 amino acids, is ubiquitous, regulates the expression of thousands of genes, and is necessary for life. In GR-β, on the other hand, the hormone-binding region is so short that it does not allow binding to the GC; it is localized in the nucleus, it is inactive, and it functions as a dominant negative. The different isoforms are then translated from multiple translation start sites, and undergo various post-translational modifications, which generate an additional level of complexity of GR biology [12,15].

## 3. GILZ in the Immune System

GILZ was first reported as a gene induced by synthetic GC dexamethasone and was found in murine lymphocytes from the thymus, lymph nodes, and spleen [23]. A human homologue of murine GILZ protein was shown in circulating T cells and monocytes with 97% identity in amino acid sequence [24]. A large body of evidence demonstrates that GILZ is a GC-induced downstream effector, one of the earliest transcriptional targets of the activated GR. Hence, GILZ, by mediating the effects of GCs, has anti-inflammatory properties upon innate and adaptive immune cells [25]. GILZ controls essential transcription factors and cellular pathways involved in the inflammatory/immune process by physically interacting with NF-κB, c-Jun/c-Fos heterodimer, Raf-1, and Ras, among others [26,27,28,29,30]. At the cellular level, GILZ mediates typical GCs effects such as regulation of thymocyte and lymphocyte survival, neutrophil migration, macrophage activation, dendritic cell maturation, and B-cell homeostasis [25,31,32,33,34,35,36,37]. As well as GCs, GILZ supports T-cell differentiation toward an anti-inflammatory phenotype through the inhibition of T helper type-1 and type-17 proliferation and the increasing response of T helper type-2 and T regulatory cells [38,39,40,41,42].

Moreover, other members of the nuclear receptor superfamily, in addition to the GR, are involved in the modulation of immune response, inflammation and GILZ regulation. Activation of peroxisome proliferator-activator receptor alpha (PPAR-α) leads to anti-inflammatory effects due to the reduction in immune cell tissue infiltration and expression of pro-inflammatory cytokines, chemokines, and cell adhesion molecules [43,44]. Further, PPAR-α contributes to the transcriptional upregulation of GILZ mediated by GCs [45]. The renal mineralocorticoid receptor represents a third nuclear receptor involved in GILZ modulation. Evidence shows that GILZ is upregulated by aldosterone in the kidney [46]. Both GILZ and serum/GC regulated kinase 1 (the protein that participates in the control of inflammation) were among the five direct target genes of mineralocorticoid receptor activation [47,48].

Similar to GR that is virtually expressed in all organs, GILZ expression is not restricted to lymphoid tissue. It has been implicated in the regulation of the inflammatory response in a variety of cells in the myocardium, skeletal muscle, brain, kidney, adipose tissue, testis, and pulmonary epithelium and endothelium [24,49,50,51,52].

## 4. GCs in Cardiovascular Physiology and Diseases

In mammals, a remarkable GC rise occurs late in gestational time, and it is essential for fetal maturation and the development of many organs in preparation for extra-uterine life [53]. Hence, GCs are routinely administered to women at risk of preterm birth to accelerate fetal maturation and improve neonatal outcome [54]. Although prenatal GC treatment has been reported to promote growth responses of cardiomyocytes and vascular structures [55,56], the direct effect on structural and functional maturation of the cardiovascular system remains scarcely understood. Some insight into the role of GC signaling in late-gestation cardiovascular maturation comes from in vivo studies conducted on GR-knockout mice, in which structural and biochemical myocardial alterations with severe functional impairment were found [57,58]. Notably, both global and heart (cardiomyocytes and vascular smooth muscle cells) deficiency in GR produced similar abnormalities, including disorganized myofibrils and irregular cellular alignment. However, myocardial dysfunction, as far as diastolic performance is concerned, is more evident when GR disruption occurs at the whole-body level [57]. These data suggest that local GC signaling is essential to elicit physiological heart maturation. Additional evidence shows the importance of GC signaling in maintaining normal cardiovascular function in the young heart. Mice, in which GR was specifically deleted in cardiomyocytes, showed a reduction in systolic function and increased hypertrophy at the age of three months. Microarray analysis revealed dysregulated expression of genes associated with cardiomyocyte contractility, cardiac hypertrophy, and heart failure, such as dystrophin, ryanodine receptor 2, Kruppel-like factor 15, and the lipocalin-type prostaglandin D synthase. Ultimately, GR deficiency leads to premature death in both male and female mice [58]. Interestingly, using RNA sequencing it has been shown that GILZ is one of the primary targets of GR in fetal cardiomyocytes [59].

In addition to the contribution to heart maturation, GCs are also involved in the pathogenesis of cardiovascular diseases. Clinically, circulating GCs are an independent risk factor for cardiovascular diseases, and GC-dependent adverse effects such as diabetes, obesity, and hypertension impair the cardiovascular stress response [7,11,60,61,62,63,64]. Endocrine disorders with high and low levels of GCs involve cardiovascular complications. In the heart, GCs regulate a cluster of cardiomyocyte genes important for cell survival and function and for repressing the hypertrophic program [58,65,66]. GCs are also known to affect vascular remodeling by regulating a vast array of signaling pathways that include oxidative stress, vascular inflammation, and nitric oxide biosynthesis [67,68]. The inhibition of the expression of the vascular endothelial growth factor gene in vascular smooth muscle cells marks their capacity as anti-angiogenic agents [69,70]. Interestingly, the activation of GR in adult cardiomyocytes is required for the maintenance of the T-tubule system. The remodeling of these ultrastructures that are the extensions of cellular membrane that penetrate into the cell, permitting a rapid transmission of the action potential, is considered a potential target in heart failure [71]. GCs signaling seems crucial for T-tubule formation also in human-induced pluripotent stem cell-derived cardiomyocytes, and this can be relevant to the challenging issue of cell maturation that is one of the barriers for the cell therapy in the heart [72].

## 5. GILZ in the Cardiovascular System

### 5.1. Chronic Inflammation

The experimental and clinical studies that link the innate and adaptive immune systems to the pathogenesis of heart failure have grown exponentially since the original description in 1990, of the ongoing inflammatory response in patients with chronic heart failure. Pro-inflammatory cytokines (tumor necrosis factor alpha (TNF-α), interleukin-1β (IL-1β), IL-6, IL-8, IL-17, and IL-18), detected in the heart of patients with heart failure (but not in non-failing hearts), can induce compensatory cardiac hypertrophy and fibrosis in the setting of cardiac injury [73,74,75]. The quantity of circulating cytokines positively correlates with adverse cardiovascular outcomes and prognosis [76]. On the other hand, a wide array of immune cells (monocytes, macrophages, T lymphocytes, B lymphocytes, and neutrophils) has been observed in the hearts from patients with heart failure in the absence of discernible myocardial injury or infection [49,51,73,77]. Increased recruitment of immune cells to failing myocardium contributes to adverse remodeling and myocardial dysfunction [10,78]. Macrophages, for instance, are centrally involved in inflammatory tissue remodeling, resolution of inflammation after myocardial infarction and left ventricular remodeling [79]. The function of macrophages in the heart is largely unknown, and we can hypothesize that GILZ expression in macrophages could affect their function in the steady state or in the genesis of heart failure. Indeed, it has been previously shown that downregulation of GILZ expression increases macrophage activation [80].

Chronic inflammation is one of the essential processes in the pathogenesis of heart failure, traditionally a “non-inflammatory” disease, and represents an independent cardiovascular risk factor [81]. While the importance of GC signaling and GILZ in triggering inflammatory cascade and sustaining low-grade chronic inflammation is compelling in the cardiovascular context, at least equally fascinating is the question of how GCs and GILZ impact non-inflammatory myocardial cells (cardiomyocytes, coronary vascular cells) in the pathological condition. The main pieces of research, discussed below, are listed in Table 1.

### 5.2. Vascular Dysfunction

The activation and dysfunction of the endothelium in response to pathologic stimuli is a well-recognized component of the evolution of heart failure syndrome. The dynamic interface between microvasculature and immune cells relies on the upregulation of surface molecules to interact with leukocytes and platelets, in order to capture leukocytes from the bloodstream and trigger their extravasation and recruitment, thus targeting inflammation to specific tissues [82]. Another implication of the continuous release of pro-inflammatory cytokines is the development of endothelial dysfunction, linked to a deficit in eNOS function and nitric oxide bioavailability, hallmarks of hypertension, heart failure and chronic conditions with a prominent vascular component such as diabetes and aging [83,84]. Inflammatory activation of endothelial cells also plays a central role in the accumulation of inflammatory cells and lipids in the vascular wall during atherogenesis [85].

The role of GILZ in the vascular system has been investigated in several studies. The mechanism by which GILZ exerts an anti-inflammatory role in endothelial cells mainly involves NF-κB, whose inhibition may occur through different mechanisms (phosphorylation status, nuclear translocation, or cytoplasmic sequestration), resulting in downregulation of cytokines, chemokines, and cellular adhesion molecules.

In a pioneering study, microarray screening of human umbilical vein endothelial cells (HUVECs) under shear stress evaluated groups of genes involved in cellular functions, such as cell proliferation and differentiation, maintenance of vascular tone, extracellular matrix organization, and inflammatory process. Among inflammation-regulating genes with significantly altered expression, the analysis revealed an enhanced expression of GILZ [86].

Other investigations conducted on human and rodent endothelial cells have followed this early report. In the first study on the role of GILZ in human endothelial cells, overexpression of GILZ via transient transfection was associated with decreased expression of pro-inflammatory molecules. After TNF-α treatment, the levels of IL-6, and IL-8, adhesion molecules E-selectin, and intercellular adhesion molecule 1 (ICAM-1], and chemokine monocyte chemoattractant protein 1 (MCP-1) were significantly lower in GILZ-overexpressing cells compared to non-transfected cells. Through the modulation of NF-κB p65 subunit-DNA binding, exogenous GILZ exerted inhibitory effects on endothelial cell adhesive function, reducing the capacity of HUVECs to support TNFα-dependent leukocyte rolling, adhesion, and transmigration. In contrast, silencing endogenous GILZ in GC-treated and -untreated HUVECs did not alter their TNFα-induced adhesive function. The findings also indicated that the suppression of NF-κB transcriptional activity did not entirely depend on a protein–protein interaction that prevents p65 nuclear translocation. The experiments conducted in HUVECs were then performed in human microvascular endothelial cells as well; the analyses showed comparable findings with GILZ having the same impact on NF-κB-dependent transcriptional activity in human microvascular endothelial cells, which respond to GILZ in a manner consistent with the response in primary HUVECs. These results revealed a previously unrecognized potential of GILZ to inhibit the inflammatory activation of human endothelial cells, although the exact mechanism through which GILZ affects NF-κB p65 DNA binding remains unclear [87].

A second study investigated the role of GILZ in human healthy saphenous veins and degenerated aortocoronary saphenous vein bypass grafts obtained from patients undergoing coronary bypass surgery. A significant reduction of GILZ mRNA and protein expression in degenerated aortocoronary saphenous veins was accompanied by a marked increase in inflammatory markers Toll-like receptor 2 (TLR2) and MCP-1. In addition, endogenous GILZ function was assessed in HUVECs. Under pro-inflammatory condition (TNF-α treatment), both GILZ mRNA and protein levels were downregulated whereas TLR2 and MCP-1 were upregulated; contrarily, laminar shear stress (in an anti-inflammatory and anti-atherosclerotic setting) induced GILZ mRNA and protein elevation. Mechanistically, modulation of GILZ expression in HUVECs involved p38 mitogen-activated protein kinase and ZFP36 (known to destabilize GILZ mRNA in macrophages). Finally, to determine functional implications of GILZ downregulation, GILZ gene was silenced by siRNA and the NF-κB activation pathway was assessed. In GILZ knockdown HUVECs, NF-κB activation and p65/p50 subunit nuclear translocation were increased when compared to control transfected cells. Moreover, TNF-α promoted upregulation of TLR2, ICAM-1 and E-selectin in HUVECs lacking GILZ. These data showed that the absence of GILZ drives a pro-inflammatory response and that its modulation might be a critical step in vascular adverse remodeling and atherogenesis [88].

An additional mechanism regarding GILZ-induced NF-κB inhibition was determined in rat primary retinal microvascular endothelial cells using lentivirus-mediated GILZ overexpression or silencing. In pathological conditions, these cells secrete pro-inflammatory cytokines, which promote leukocyte adhesion, undermine the retinal vascular barrier integrity, and ultimately lead to inflammatory retinopathy and neuronal damage. The study established that GILZ overexpression inhibited NF-κB p65 nuclear translocation in retinal microvascular endothelial cells with lipopolysaccharide stimulation. Interestingly, NF-κB signaling shutdown did not involve the degradation of inhibitory κB but was associated with enhanced p65 dephosphorylation (at Ser536) that, in turn, is responsible for the downregulated ICAM-1 and MCP-1 expression. In contrast, GILZ silencing resulted in significant increases in ICAM-1 and MCP-1 expression after lipopolysaccharide. The regulatory effects of GILZ on retinal microvascular endothelial cells were confirmed by analysis of retinal inflammation in vivo. Intravitreal injection of lipopolysaccharide decreased retinal GILZ and increased at the same time ICAM-1 and MCP-1 expression; contrarily, in GILZ-overexpressing retinas, these increases were markedly attenuated [89].

Another demonstration of GILZ involvement in the modulation of vascular inflammation is shown in a study conducted in patients with Sjögren’s syndrome, a systemic autoimmune disease of salivary and lacrimal glands, as well as salivary glands of non-obese diabetic mice (a model of Sjögren’s syndrome-like disease). First, a marked difference in leukocyte infiltration was observed in the salivary glands of non-obese diabetic mice, compared to control mice, and in biopsy samples of subjects with a diagnosis of Sjögren’s syndrome compared to healthy subjects. Second, immunofluorescent images showed a reduced expression of GILZ in both non-obese diabetic mice and Sjögren’s syndrome patients with respect to their respective controls. The reduced expression of GILZ was associated with a significant increase in the pro-inflammatory cytokine level, IL-17, and a decrease in developmental endothelial locus 1 (Del-1) expression that serves as an endogenous negative regulator of leukocyte adhesion and transmigration into inflamed tissues. Finally, in vitro IL-23 treatment of mouse salivary gland cells induced a significant reduction in Del-1-positive cells along with a marked increase in IL-17-positive cells. Interestingly, co-culture with GILZ-expressing mesenchymal stem cells significantly reversed the pro-inflammatory phenotype of salivary gland cells, abrogating the impact of treatment with IL-23 [90].

Taken together, these findings raise the possibility that induction of endothelial GILZ expression could represent a potential goal for the treatment of the inflamed endothelium. Increasing knowledge of the anti-inflammatory action of GILZ in endothelial cells lays out the new mechanistic background for the targeting of pathological leukocyte recruitment, and ideally, may not only attenuate micro- and macrovascular dysfunction but also interfere with the initial step of several chronic pathologies.

### 5.3. Myocardial Damage and Remodeling

In 2013, the first study described the ability of GCs to induce GILZ expression in the myocardium and cardiac cells (primary cultured rat cardiomyocytes and H9c2 cell line). C57BL/6 mice were administered intra-peritoneally with dexamethasone or vehicle to address GILZ induction in the myocardium. Mice treated with dexamethasone showed elevated levels of GILZ protein compared to the control group. At the cellular level, GILZ upregulation after GC challenge occurred in both primary rat cardiomyocytes and H9c2 cells with a similar dose- and time-dependent pattern. The blockade of the GR resulted in the absence of GILZ induction after dexamethasone, suggesting the necessity of GR activation for the modulation of GILZ. The evaluation of signaling involved in GC-dependent GILZ induction revealed the role of PKA and p38 MAPK pathways that negatively regulated GILZ expression, as shown by the increase of GILZ expression after exposing cardiomyocytes to p38 or PKA inhibitors [91].

Further insight into the role of GILZ in the heart emerges from the data showing that GILZ mediates GC-dependent cytoprotection. H9c2 cells pre-treated with corticosterone were protected from doxorubicin-induced apoptosis. siRNA-mediated knockdown of GILZ reversed the beneficial effect of corticosterone on doxorubicin-dependent caspase activation. Conversely, GILZ overexpression significantly reduced the number of apoptotic cells and increased cell survival. These findings implied that GILZ is essential for GC-mediated protection. The underlying mechanism of cytoprotection entailed the elevation of Bcl-xL, a pro-survival Bcl2 family member. The overexpression of Bcl-xL protein in the absence of Bcl-xL mRNA modulation likely involves a post-transcriptional mechanism. The physical interaction of Bcl-xL and GILZ, as reported for other proteins (NF-κB, Raf, Ras) remains to be determined [92].

The possible involvement of GILZ in the regulation of cell death machinery emerges from acute cryoinjury experiments demonstrating a marked reduction in cardiac GILZ three hours after damage induction. This study also showed that GILZ-overexpressing mesenchymal stem cells injected into the damaged area immediately after cryoinjury have a higher potential to increase in regulatory T cells and IL-10-positive cells, and decrease Th-17 cells, apoptotic and necrotic cells [93].

The most recent study addressing the role of GILZ in cardiac pathophysiology was conducted in GILZ-knockout mice undergoing angiotensin II-releasing pump implantation to induce cardiac hypertrophy and diastolic dysfunction. Firstly, in wild-type animals, chronic infusion of angiotensin II resulted in the upregulation of myocardial GILZ mRNA and protein, strengthening the potential significance of GILZ in cardiovascular pathologies. Unexpectedly, several aspects of angiotensin II-mediated detrimental effects were similar in wild-type and GILZ-knockout mice. The fibrotic response to angiotensin II was similar in both groups suggesting the lack of a significant involvement of GILZ in the development of myocardial fibrosis upon chronic angiotensin II excess. Similarly, the upregulation of inflammatory genes in the heart after angiotensin II infusion was not aggravated by GILZ ablation. Despite these similarities, following angiotensin II infusion, GILZ-knockout mice had a significantly more profound diastolic dysfunction and a thicker left ventricular wall. These facts suggested the presence of cardiomyocyte subcellular changes that might have been responsible for the deficit in ventricular relaxation and myocardial stiffness. Indeed, cardiomyocyte hypertrophy was more severe, and it could explain the enhanced deterioration of diastolic function in GILZ-knockout hearts. Mechanistically, this study suggests that GILZ may interact with FoxP3 and GATA4 in the development of cardiac hypertrophy. FoxP3 and GATA4 were markedly upregulated after Ang II, but in the hearts of GILZ-knockout mice, such an increase was less pronounced (for FoxP3) or absent (for GATA4) [94]. Although the details of molecular interplay of GILZ with FoxP3 or GATA4 in cardiomyocytes remain to be determined, the absence of GILZ may condition their expression, leading to the excessive hypertrophic response. This previously unknown role of GILZ in hypertrophic growth can be of great interest from a translational point of view because LV hypertrophy is a risk factor for heart failure and sudden death and represents a valid therapeutic target.

## 6. Conclusions

**Figure 1:**
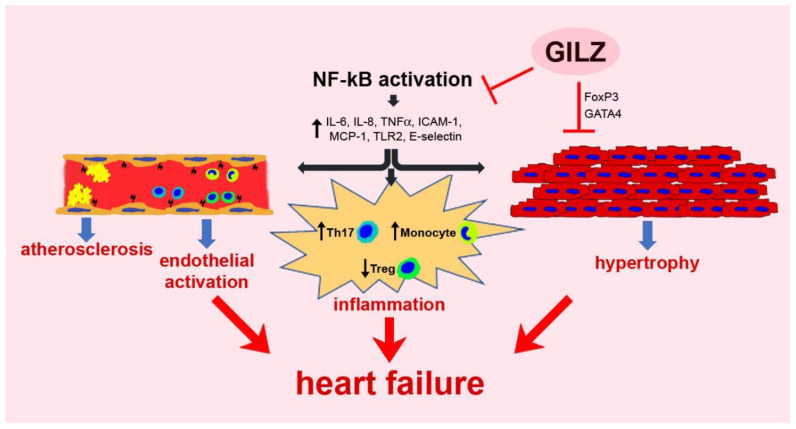
A schematic representation of GILZ participation in pathologic processes regarding the myocardium and the vascular system.

The relevance of GILZ in cardiovascular biology remains mostly unknown. However, despite the limited availability of studies, it is becoming evident that GILZ participates in several critical processes that, in various scenarios, contribute to alterations in the myocardium and the vascular system (Figure 1). Further research focusing on molecular mechanisms regulated by GILZ in the cardiovascular system is warranted to understand better the pathophysiological background, and conceive new therapeutic opportunities based on targeting GILZ signaling.

To date, the approaches for the in vivo delivery of GILZ-based molecules (i.e., cell-permeable recombinant GILZ protein) have been tested in pre-clinical studies for a variety of conditions with underlying dysregulation of immune and inflammatory mechanisms, but although promising, such therapies have not entered clinical trials. Furthermore, because of the well-known but yet-to-be addressed harmful effects of prolonged use of GCs, research on GILZ in a cardiovascular context needs to continue.

## Figures and Tables

**Table 1:** Developmental endothelial locus 1; GILZ, glucocorticoid-induced leucine zipper; HUVECS, human umbilical vein endothelial cells; ICAM-1, intercellular adhesion molecule 1; IL-, interleukin-; LPS, lipopolysaccharide; MCP-1, chemokine monocyte chemoattractant protein-1; NF-κB, nuclear factor-κB; RMECs, retinal microvascular endothelial cells; TLR2, Toll-like receptor 2; TNF-α, tumor necrosis factor α; Tregs, regulatory T cells.

In Vitro/In Vivo Model	Treatment	Results	Reference
HUVECs(GILZ overexpression)	TNF-α	Reduction of IL-6, IL-8, E-selectin, ICAM-1, MCP-1	[87]
HUVECs(GILZ silencing)	TNF-α	No effect	[87]
HUVECs(GILZ silencing)	TNF-α	Enhancing of NF-κB nuclear translocation; elevation of TLR2, ICAM-1, E-selectin	[88]
RMECs(GILZ overexpression)	LPS	Inhibition of NF-κB nuclear translocation; reduction of ICAM-1, MCP-1	[89]
RMECs (GILZ silencing)	LPS	Elevation of ICAM-1, MCP-1	[89]
Mouse salivary gland cells	IL-23	Inhibition of GILZ; elevation of IL-17; reduction of Del-1	[90]
Primary rat cardiomyocytes, H9c2 cells	Dexamethasone	Elevation of GILZ	[91]
C57BL/6 mice	Dexamethasone	Elevation of myocardial GILZ	[91]
H9c2 cells(GILZ overexpression)	Doxorubicin	Decreased apoptosis	[92]
H9c2 cells(GILZ silencing)	Doxorubicin	Increased apoptosis	[92]
Balb/C mice(myocardial infarction)	Injection of GILZ-overexpressing MSCs	Elevation of Tregs, IL-10^+^ cells; reduction of IL-17^+^ cells, necrotic cells	[93]
GILZ-knockout mice	Angiotensin II infusion	Increased cardiomyocyte hypertrophy, diastolic dysfunction	[94]
